# Psychosis and Silent Celiac Disease in a Down Syndrome Adolescent: A Case Report

**DOI:** 10.1155/2011/970143

**Published:** 2011-12-01

**Authors:** Amparo Morant

**Affiliations:** Clínica de Neuropediatría, Dra. Amparo Morant, Calle Jesús 101-7^a^, 46007 Valencia, Spain

## Abstract

Celiac disease is an autoimmune systemic disorder. It presents gastrointestinal and nongastrointestinal manifestations as well as associated conditions. We report a 16-year-old Down syndrome girl who presented psychosis symptomatology, and she was diagnosed as having silent celiac disease. Olanzapine treatment and gluten-free diet were satisfactory. It is necessary to consider celiac disease in Down syndrome patients with psychiatric symptoms, mainly psychotic symptomatology.

## 1. Introduction

Celiac disease (CD) is an autoimmune systemic disorder caused by a gluten permanent intolerance. CD mainly affects the digestive tract, but not exclusively, because it also presents nongastrointestinal manifestations and associated conditions, which are unlikely to show accompanying digestive symptoms; then it is called silent CD [1, 2]. 

Down syndrome is one of these associated conditions because it presents an increase of autoimmune diseases frequency, especially CD and hypothyroidism [1, 3, 4]. 

On the other hand, CD has also been linked to various neurological and neuropsychological manifestations [5, 6]. Among them is schizophrenia although its relationship with CD is not fully established [7–10]. 

We report a 16-year-old Down syndrome girl diagnosed as having silent CD while suffering psychotic symptoms.

## 2. Patient

A 16-year-old Down syndrome girl (trisomy 21) was referred to our clinic reporting dizziness. Her family had noticed changes in her behavior over the last few months: speaking alone, sleep disturbances, irritability and emotional instability, school work deterioration, and social withdrawal. There was no fluctuation in her conscious level. Toxic ingestion matter was negative, and no digestive manifestations were presented. 

The physical examination showed a specific phenotype and mild mental retardation. She was calm, collaborating and answering some simple questions quietly. Her mental state was apparently normal. She presented a minimal bilateral intention tremor and dysdiadochokinesia.

A cerebral magnetic resonance imaging (MRI) revealed a discreet atrophy of the cerebellar vermis. The electroencephalogram (EEG) result was normal. Laboratory data showed macrocytosis (mean corpuscular volume: 101 fl [80–99]) without anemia, and IgA hypergammaglobulinemia level of 646 mg/dL (70–400) and IgG level of 1306 mg/dL (700–1200). Vitamin B6 and folic acid were normal. T4 and TSH were normal. 

IgA gliadin antibodies were 38.1 U/mL (positive >10), and IgG gliadin antibodies were 16.7 U/mL (positive >10). IgA antibodies to tissue transglutaminase (anti-t-TG) were 230.5 U/mL (positive >10). HLA-DQ2 phenotype was positive. Intestinal biopsy showed an atrophic duodenitis type 3B Marsh classification.

Treatment began with olanzapine (20 mg/day) along with gluten-free diet. Psychotic symptoms improved in two months. Antibodies were negative a year later.

After two years of treatment, we proceeded to phase out olanzapine over a 6-month period. Psychotic symptoms (personality change, insomnia, and auditory hallucinations) reappeared a month after drug treatment withdrawal, while the patient kept gluten-free diet correctly. Antibodies continued to be negative too. 

After restarting olanzapine treatment, psychotic symptoms disappeared within a month. She is currently asymptomatic, keeping the gluten-free diet as well as the olanzapine treatment.

## 3. Discussion

We present a Down syndrome adolescent who developed psychotic symptoms and was diagnosed as having silent CD. 

Down syndrome presents a greater frequency of autoimmune diseases. CD prevalence in Down syndrome patients is between 5 and 12 percent. Many studies recommend checking up on CD in Down syndrome patients periodically, since one-third of patients has non-gastrointestinal symptoms [1].

Psychiatric symptoms in Down syndrome patients are varied, notably depression and early dementia from the age of 40 on. Psychiatric problems diagnosis in these kind of patients tends to be quite complicated, due to mental retardation existence [11].

The relationship between CD and schizophrenia was first described in 1953 by Bender [12]. Since then, there have been different studies, and various hypotheses have been suggested (cerebral hypoperfusion, intestinal malabsorption, disorders of neurotransmitters metabolism, and autoimmune disease) although the causal relationship between schizophrenia and CD has not been established yet [6, 13].

A Down syndrome adolescent case with psychotic symptoms and CD has never been published, despite the relationship that exists between these three conditions. Only Serratrice et al. [14] described a 42-year-old Down syndrome woman who presented psychotic symptoms. She was under treatment for a year with pharmacotherapy (haloperidol, olanzapine) with no improvement. Later investigations showed positive IgA and IgG gliadin antibodies, positive HLA-DQ2, and normal intestinal biopsy. Then she was diagnosed having silent CD, and she started with a gluten-free diet. After a year with a gluten-free diet, together with olanzapine treatment, the psychotic symptoms improved clearly. 

These authors emphasize three facts: the difficulty of a differential diagnosis between psychotic symptomatology and the onset of early dementia in Down syndrome patients, the role of CD in psychiatric symptoms appearance, and the improvement of psychotic symptoms with a gluten-free diet. Their diagnosis was silent CD as even intestinal biopsy was normal. Despite this fact, we believe that silent CD should not be considered in these cases; it is better to think of a latent CD, and the role and the importance of gluten-free diet is even more controversial.

In our case, the diagnosis was a silent CD because antibodies were positive, HLA-DQ2 was positive, intestinal biopsy was type 3B, and she has not gastrointestinal symptoms. The differential diagnosis with early dementia was not considered due to patient age.

Our patient improved considerably with olanzapine treatment together with gluten-free diet. Olanzapine withdrawal was decided after two years of treatment, bearing in mind that some patients with schizophrenia and CD had improved only with the gluten-free diet [15]. In our case, olanzapine treatment had to be reestablished. This is the reason why we cannot establish a specific causal relationship between CD and psychotic symptomatology.

In conclusion, CD has to be investigated in any Down syndrome patient with psychiatric symptoms, especially psychotic type symptoms, even when there are no gastrointestinal manifestations.
